# Ultrasonography evaluation on the protective effect of combination therapy of beraprost sodium and aspirin on arteries occlusion and stiffness in patients with type 2 diabetes mellitus - a prospective, randomized study

**DOI:** 10.1186/s12902-022-01007-5

**Published:** 2022-04-02

**Authors:** Xian Lin, Yuying Chen, Wan Lu, Jin Li, Li Fu, Jingyu Yin, Meng Ren, Li Yan, Chuan Yang

**Affiliations:** 1 Department of Ultrasound, Guangdong Province Traditional Chinese Medical Hospital, 111, Dade Road, Yuexiu District, Guangzhou, Guangdong China; 2 Department of Endocrinology, Sun Yat-sen Memorial Hospital, Sun Yat-Sen University, 107, Yanjiangxi Road, Yuexiu District, Guangzhou, Guangdong PR China

**Keywords:** Beraprost Sodium, Aspirin, Type 2 Diabetes Mellitus, Atherosclerosis, Medial Arterial Calcification

## Abstract

**Background:**

Patients with type 2 diabetes mellitus (T2DM) are susceptible to developing symptomatic peripheral arterial disease (PAD). As a proven vasodilator and antiplatelet agent, the efficiency of Beraprost sodium (BPS) on the prevention of arteries occlusion and stiffness in T2DM patients with PAD has not yet been fully investigated.

**Methods:**

From July 2010 to April 2012, 64 Patients enrolled were randomly assigned to the combined therapy group (*n*=32), which received combination therapy with BPS (60 μg/day) and aspirin (100 mg/day), or to the control group (*n*=32), which only received aspirin (100 mg/day). After randomization, the patients were followed up at years 0, 1, 2, 3, 4, and 5 with the evaluation of carotid intima-media thickness (CIMT), pulse wave velocity (PWV), inner artery diameter, stenosis rate, and medial arterial calcification (MAC) of lower limb arteries via high-resolution ultrasound measurement. Adverse events were also recorded in each visit.

**Results:**

There was no significant change of the CIMT during the follow-up in both groups when compared to the baseline. Similar results were also observed in the PWV measurement. Significantly increases in the inner artery diameter of the dorsal pedal artery and posterior tibial artery were observed in patients with BPS and aspirin administration during the follow-up. Patients in the combined therapy group experienced marked improvement of MAC in the dorsal pedal artery and posterior tibial artery at the end of the follow-up. No significant difference in the adverse events was found between the combined therapy group and the aspirin group.

**Conclusion:**

The combined therapy of BPS and aspirin showed a protective effect on arteries occlusion and stiffness in T2DM patients with PAD, along with a significant improvement of inner artery diameter and MAC in lower limbs.

**Trial registration:**

http://www.chictr.org.cn, ChiCTR-TRC-10000919. Prospectively registered on 2010/06/29.

## Background

Patients with diabetes are susceptible to developing symptomatic peripheral arterial disease (PAD), which is closely related to increased mortality rate and low quality of life. The overall prevalence of PAD has been estimated to be 20% and 29% in patients with diabetes over 40 and 50 years old, respectively [1, 2]. PAD in diabetic patients is diffuse and is most severe in the crural and foot arteries, which is characterized by arterial calcification, large artery stiffness, and occlusion of the arteries [3]. Management of PAD includes exercise, smoking cessation, statin therapy, and antiplatelet therapy with aspirin or clopidogrel [4].

Beraprost sodium (BPS) is a stable orally active prostacyclin (PGI2) analog, which has an effect on the relaxation of the smooth muscle cells and vasodilation via binding to PGI2 membrane receptors and inhibiting the release of Ca^2+^ from intracellular storage sites [5]. Moreover, BPS also alleviates vascular thrombosis by inhibiting platelet aggregation. BPS has been proven to be an effective agent in the treatment of patients with pulmonary arterial hypertension and Buerger’s disease [6, 7].

Recently, it was reported that BPS reduced lower limb ischemic symptoms and carotid intima-media thickness in patients with PAD [8]. It was also found that chronic diabetic foot ulcer patients experienced a significant increase in the wound healing rate after the administration of BPS, suggesting that BPS might be a potential treatment in treating peripheral circulatory disorders in patients with diabetes [9]. However, the exact effect of BPS on arterial calcification and occlusion of peripheral arteries, especially lower limb arteries in patients with T2DM has not yet been fully investigated. Thus, the present study aimed to investigate the difference of changes in the inner diameter and medial arterial calcification (MAC) rate of lower limb arteries between T2DM patients with 5 years combination therapy of BPS and aspirin, and those received solely aspirin treatment.

## Methods

### Study participants

Enrollment criteria included: 1. Patients diagnosed with type 2 diabetes between the age of 50 and 75; 2. Those had a carotid intima-media thickness (CIMT) larger than 1.1mm via ultrasound measurement. Exclusion criteria included: 1. Serum AST, ALT, SCr≥1.5 times normal value; 2. Those had ischemic vascular events in the last 3 months, including myocardial infarction, unstable angina pectoris, stable stenocardia, cerebral arterial thrombosis, or hemorrhagic apoplexy; 3. SBP>160mmHg or DBP>100mmHg; 4. Serum HbA1c>8.0%; 5. Anti-thrombus drug and antiplatelet drug administration in the last 3 months; 6. Patients with peptic ulcer or hemorrhage of the digestive tract; 7. Drug allergy in prostacyclin synthase or non-steroidal anti-inflammatory drugs; 8. Patients who are in suckling period or pregnant and having pregnancy planning.

### Study design

This single-center, prospective randomized study was conducted in the Endocrinology Department of Sun Yat-sen Memorial Hospital, Sun Yat-sen University from July 2010 to April 2012. The study was conducted in accordance with the ethical principles of the Helsinki declaration and was approved by the institutional review board of Sun Yat-sen Memorial Hospital, Sun Yat-sen University. The study has been registered in the Chinese clinical trial registry center (ChiCTR-TRC-10000919, 29/06/2010). Signed informed consent was obtained from all the patients. We used the CONSORT checklist when writing our report.

All the participants enrolled were randomly divided into combination therapy group and aspirin group with a ratio of 1:1. A list of random numbers was generated by an independent statistician and then used to randomly allocate the treatments to those two groups. The randomization code list was generated by a third party, the medical statistics support team of Sun Yat-sen University.

**Fig. 1:**
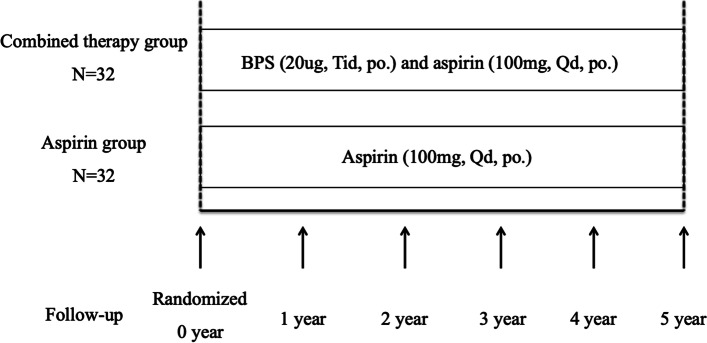
Study design. 64 patients were randomly assigned to the combined therapy group (32 patients), which received combination therapy with BPS (60 μg/day) and aspirin (100 mg/day), or to the control group (32 patients), which only received aspirin (100 mg/day). After randomization, the patients were followed up at year 0, 1, 2, 3, 4, and 5. BPS: beraprost sodium

After randomization, the participants will take either a combined BPS (20ug, Tid, PO. Dorner) and aspirin (100mg, Qd, PO.) treatment or solely aspirin (100mg, Qd, PO.) treatment during the follow-up. Demographic data as well as blood pressure, levels of blood glucose, and biomedical indexes of liver, kidney function, and medication use were collected in the first visit. CIMT, Pulse wave velocity (PWV), inner artery diameter, stenosis rate, and MAC of lower limb arteries were measured before the intervention and annually thereafter. The presence of adverse events, such as bleeding, headache, hot flushes, shock, interstitial pneumonia, abnormal liver function, hypersensitivity reaction, angina, and gastrointestinal discomfort were also recorded in each visit. Drug compliance was evaluated via telephone follow-up by a qualified staff of our department. The study algorithm was shown in Figure 1.

### Outcome measurements

#### CIMT

CIMT is a measure of the combined thickness of intima and media layers of carotid artery, and the increase in CIMT was proven to be the factor responsible for the development and progression of atherosclerosis [10]. In the present study, CIMT was measured using high-resolution ultrasound according to the standardized protocol recommended by the American Society of Echocardiography, and CIMT≥1.1mm was defined as a marker of atherosclerosis of carotid [11].

#### PWV

PWV is defined as the velocity at which the blood pressure pulse propagates through the circulatory system, usually an artery or a combined length of arteries. PWV was commonly used to evaluate arterial stiffness, which was predictive in future cardiovascular events and all-cause mortality independent of conventional cardiovascular risk factors [12]. In the present study, the measurement of the brachial artery to ankle artery PWV was conducted to obtain baPWV.

#### Other artery parameters of the lower limbs

The inner diameter of the dorsal pedal artery and posterior tibial artery was measured at their origin in US measurement, which is a useful index for the evaluation of arteries occlusion. The narrowest diameter of the stenosis (A) in dorsal pedal artery and posterior tibial artery was compared with the inner diameter at their origin (B), and the stenosis percentage was calculated via (1−A/B)×100% [13]. The presence of uniform, smooth, linear, and non-stenotic calcified arterial wall in the US assessment was diagnostic of MAC [14].

#### Statistics analysis

All quantitative data were presented in mean ± standard deviation (SD). Multiple comparisons of changes in CIMT, PWV, and the inner diameter of dorsal pedal artery and posterior tibial artery between different time points were analyzed using analysis of variance for repeated measurements with Bonferroni’s correction. The difference between ratios was analyzed via Pearson's nonparametric chi-square test and Fisher exact test. The differences of the demographic data between the combined therapy group and aspirin group were analyzed via Two-sided Student's t-test. A *P* value of <0.05 was considered to be significant. SPSS 20.0 (SPSS Headquarters, Chicago, IL, USA) and SAS 9.4 (SAS Institute Inc, Cary, NC, USA) were used to carry out the statistical analysis.

## Results

**Fig. 2:**
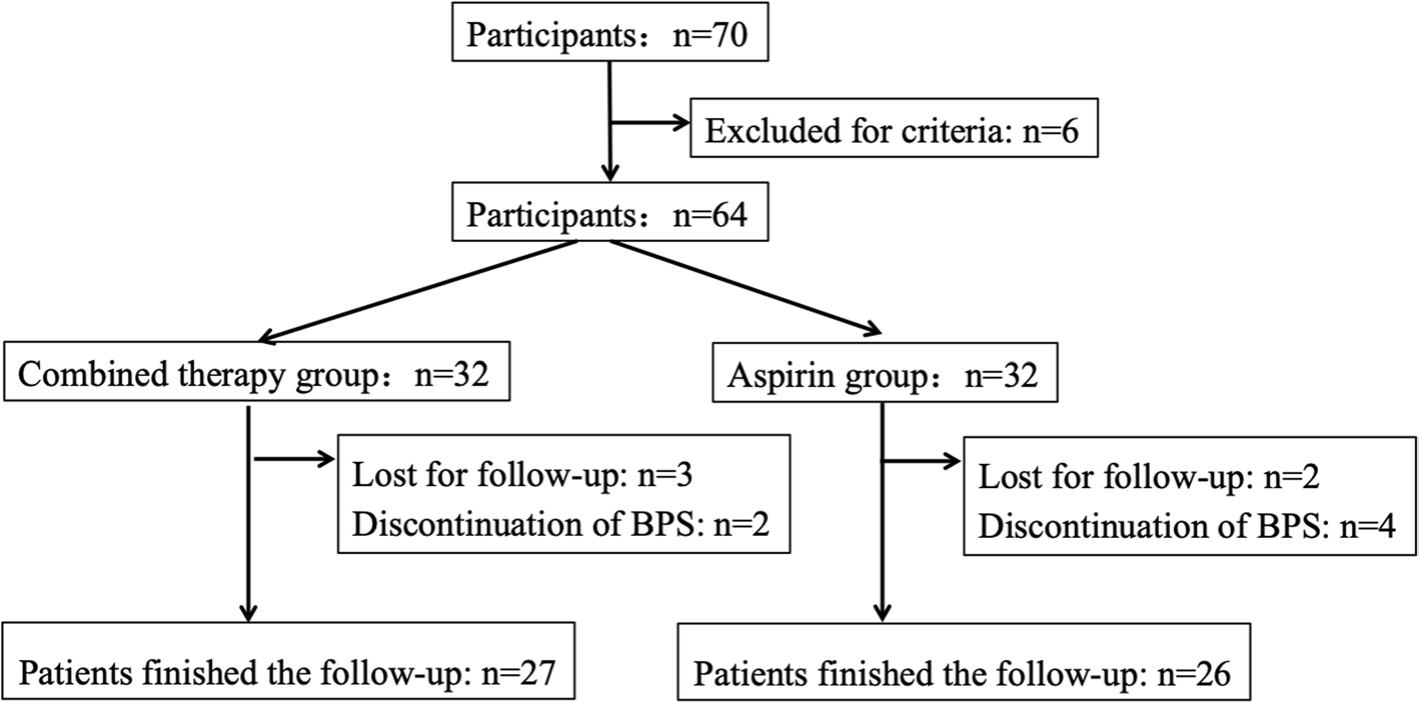
Study enrollment. A total of 64 subjects were initially enrolled. 5 patients in combined therapy group and 6 patients in aspirin group were lost to the follow-up for personal reasons or discontinuation of BPS. The present study analyzed 27 patients in combined therapy group and 26 patients in aspirin group

From July 2010 to April 2012, a total of 64 patients were enrolled according to the inclusion and exclusion criteria, with 32 patients in each group. At the end of the study, 5 patients in combined therapy group and 6 patients in aspirin group were lost to the follow-up for personal reasons or discontinuation of BPS. Thus, 27 patients in combined therapy group and 26 patients in aspirin group completed this 5 years follow-up with intact data (Fig. 2).

Table 1 shows the baseline characteristics of the study subjects before BPS treatment. The two groups did not differ significantly in any baseline characteristics (age, sex, BMI, SBP, DBP, renal function variables, diabetes-associated variables, or concomitant medication). At the end of the follow-up, most variables still had no significant difference between the two groups except SBP (*P*=0.044) and AST level (*P*=0.011), which were shown in Table 2.

**Table 1:** Demographic data collected at the baseline

Variables	Combined therapy group (*n*=32)		Aspirin group (*n*=32)		*P*
	Mean	Std	Mean	Std	
Age (year)	62.7	6.4	64.4	6.5	0.338
Male Sex (%)	63.0%	/	65.4%	/	0.541
BMI (kg/m^2^)	24.71	3.12	24.03	3.28	0.442
SBP (mmHg)	142.56	15.92	149.05	15.41	0.138
DBP (mmHg)	74.90	8.04	77.94	7.48	0.160
HbA1C (%)	6.93	0.77	6.91	0.67	0.928
FBG (mmol/L)	6.99	2.76	6.62	1.67	0.556
CHOL (mmol/L)	5.12	0.85	5.21	0.91	0.722
TG (mmol/L)	1.52	0.68	2.02	1.47	0.118
HDL-C (mmol/L)	1.34	0.31	1.39	0.34	0.644
LDL-C (mmol/L)	3.09	0.76	2.91	0.95	0.446
AST (U/L)	20.74	4.43	27.88	16.11	0.031
ALT (U/L)	17.00	8.22	25.62	18.07	0.029
BUN (mmol/L)	6.96	1.87	6.53	1.89	0.407
CREA (μmol/L)	108.63	21.09	106.04	25.10	0.685
CCR (ml/min)	56.10	12.98	55.99	16.80	0.980
Medication use					
Antihypertensive drugs (%)	90.63	/	90.63	/	1.000
Insulin (%)	68.75	/	65.63	/	0.790
Statins (%)	15.63	/	18.75	/	0.740

Plus-minus values are mean±S.D

**Table 2:** Demographic data collected at the end of the follow-up

Variables	Combined therapy group (*n*=27)		Aspirin group (*n*=26)		*P*
	Mean	Std	Mean	Std	
BMI (kg/m^2)^	24.35	3.40	23.62	3.32	0.429
SBP (mmHg)	142.00	13.65	152.36	22.00	0.044*
DBP (mmHg)	74.70	7.60	79.93	12.53	0.071
HbA1C (%)	7.27	1.29	7.06	1.05	0.522
FBG (mmol/L)	7.58	2.04	6.90	1.43	0.169
CHOL (mmol/L)	4.55	1.14	4.93	1.03	0.209
TG (mmol/L)	1.60	1.09	1.93	1.44	0.350
HDL-C (mmol/L)	1.16	0.31	1.23	0.34	0.491
LDL-C (mmol/L)	2.90	0.80	3.14	0.79	0.274
AST (U/L)	17.26	4.64	23.65	11.62	0.011*
ALT (U/L)	18.04	7.71	22.69	16.78	0.198
BUN (mmol/L)	6.67	2.23	6.60	2.62	0.921
CREA (μmol/L)	101.85	28.56	99.84	30.87	0.807
CCR (ml/min)	57.35	17.79	55.76	21.69	0.771
Medication use					
Antihypertensive drugs (%)	37.5	/	21.9	/	0.171
Insulin (%)	21.9	/	9.4	/	0.302
Statins (%)	9.4	/	3.1	/	0.613

Plus-minus values are mean±S.D. * indicated *p*<0.05

5 patients in the combination therapy group had previous history of diabetic foot ulcers, and 2 of them required readmission for eczema and delayed wound healing in the foot, respectively. The symptoms of numbness, coldness, and intermittent claudication were markedly improved after the intervention in combined therapy groups. 3 patients in the aspirin group reported that they didn’t experience improvement of numbness and coldness in lower limbs. No diabetic foot ulcer was reported in both groups during the follow-up. Details of the adverse events were shown in Table 3. No significant difference of the adverse events was found between the combined therapy group and aspirin group.

**Table 3:** Adverse events

Adverse events	Combined group (*n*=27)	Aspirin group (*n*=26)	*P*
Heard failure	1	0	
Coronary disease	0	1	1.000
Cerebral hemorrhage	0	1	1.000
Lacunar infarction	1	0	1.000
Bellyache	1	1	1.000
Subcutaneous hemorrhage	1	0	1.000
Upper gastrointestinal bleeding	1	1	1.000
Cancer	1	2	1.000
Others^a^	6	6	1.000

^a^Others indicated total knee arthroplasty, renal calculus, et al

**Fig. 3:**
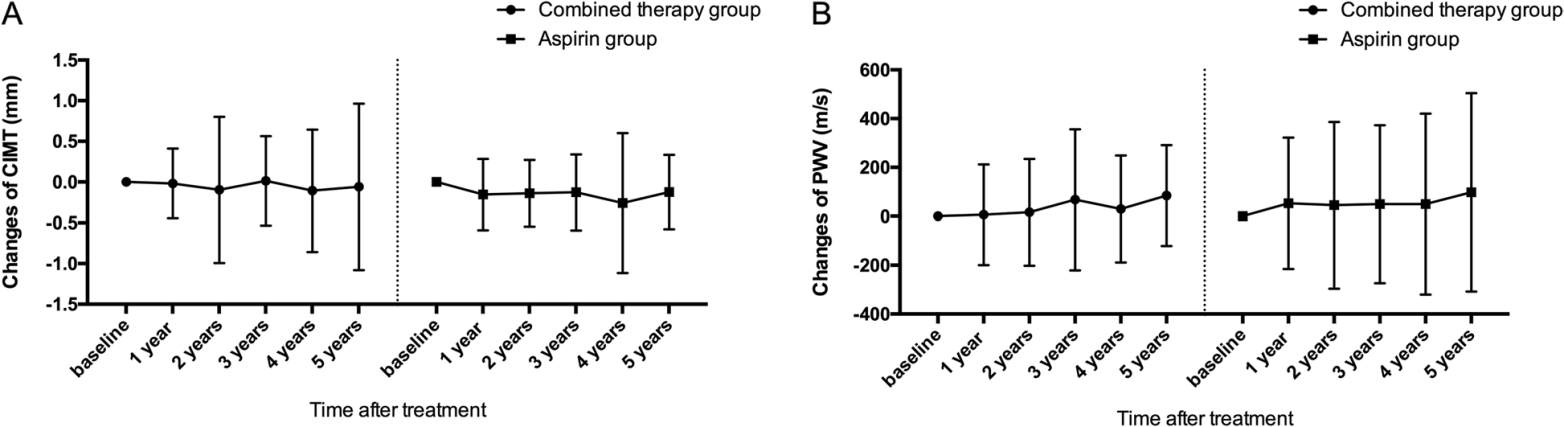
Changes of CIMT (**A**) and baPWV (**B**) in both groups during the follow-up. CIMT: carotid intima-media thickness. baPWV: measurement of brachial artery to ankle artery PWV

There was no significant change of the CIMT during the follow-up in both groups when compared to the baseline (Fig 3A). The two groups did not differ significantly in the changes of the CIMT at the end of the follow-up (-3.4% vs -7.6%, *P*>0.05). Similar results were also observed in the PWV measurement (Fig 3B). The two groups did not differ significantly in the changes of the PWV at the end of the follow-up (-5.4% vs -5.4%, *P*>0.05).

**Fig. 4:**
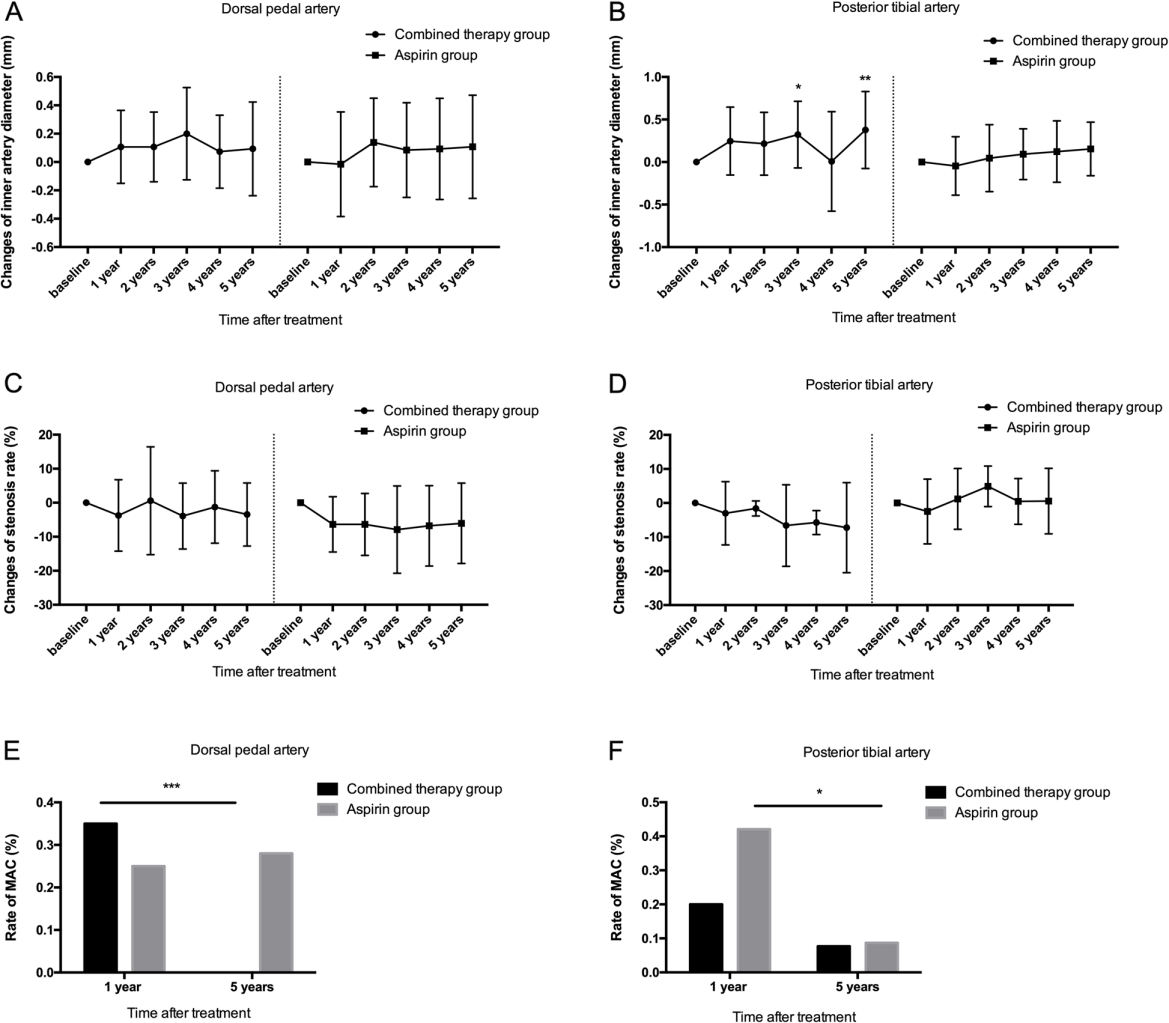
Serial changes from baseline to 5 years after intervention in inner artery diameter of dorsal pedal artery (**A**) and posterior tibial artery (**B**), stenosis rate of dorsal pedal artery (**C**) and posterior tibial artery (**D**), and rate of MAC of dorsal pedal artery (**E**) and posterior tibial artery (F) in the combined therapy group and control group. * indicated *P*<0.05, ** indicated *P*<0.01 and *** indicated *P*<0.001 when compared with the baseline. MAC: medial arterial calcification

Increases of the inner artery diameter of dorsal pedal artery and posterior tibial artery were observed in patients with BPS and aspirin administration during the follow-up, and the changes of inner artery diameter of posterior tibial reached a significant level in the 3rd (+13.4%, *P*<0.01) and 5th (+15.7%, *P*<0.001) year after BPS treatment (Fig 4A-B). No significant change of the inner artery diameter of dorsal pedal artery and posterior tibial artery was found in aspirin group during the follow-up. The stenosis rate of the former-mentioned arteries remained stable during the follow-up in both groups with no significant changes was found (Fig 4C-D). Regarding the rate of MAC, patients in combined therapy group experienced marked improvement in the dorsal pedal artery (*P*<0.001, Fig 4E) and posterior tibial artery (*P*<0.05, Fig 4F) at the end of the follow-up, when compared to the control group.

## Discussion

In the present study, we found that BPS and aspirin combined therapy showed extra advantages than aspirin treatment in the improvement of arteries occlusion and stiffness in lower limbs, which indicated that BPS might be an effective option of PAD treatment in diabetic patients. To the best of our knowledge, there are several studies [15–17] that have investigated the efficiency of the BPS on subjective symptoms and ankle pressure index in diabetic patients with PAD. However, we suggest that there are still differences between previous studies and ours. Firstly, our study is a prospective, randomized control study, which has a higher level of evidence than cohort studies [15, 16]. Secondly, our studies focused more attention on the efficiency of the BPS on ultrasound-measured arteries occlusion and stiffness, which are important supplementals to subjective symptoms for the evaluation of PAD progression. Lastly, our study has a longer follow-up time (>5 years) than the formerly mentioned studies, which provided evidence for long-term use of beraprost sodium. It is also important to note that prostanoids (including BPS) are recommended as a medical treatment for patients with PAD only when there are contraindications for revascularization or the revascularization has failed, but not as the first-line treatment according to the recent guideline [18].

A retrospective study including 188 patients showed that there were significant decreases of CIMT in patients with diabetes mellitus complicated by chronic arterial obstruction after six months of BPS administration (120mg/d), indicating that it is useful in treating peripheral circulatory disorders in such patients [16]. In the present study, we found that there was no significant difference of the changes during the follow-up when compared to the baseline. We supposed that the inconsistency between the former-mentioned studies and ours might largely attribute to the dosage variation of BPS (60mg/d vs. 120mg/d) in the two studies. Another study with a prospective design obtained similar results with ours, they found that there was no significant change of CIMT in diabetic patients after 3 years of BPS treatment, while there was a markedly increase in the control group [19]. In regard to the inner artery diameter, the useful index for evaluating the severity of atherosclerosis in late PAD patients, the present study found that patients in the combined therapy group experienced significant improvement of posterior tibial in the 3rd and 5th year after intervention. A previous animal model study also got similar results with ours; they found that there was a significant preservation of the luminal diameter in the BPS groups as compared with that in the control group after the angioplasty on rabbit [20]. Taken together, we suggested that BPS did show a protective effect on preventing PAD deterioration and subsequent arteries occlusion. And it was proposed that the protective effect of BPS on arteries occlusion might attribute to endothelial protection, inhibition of release of inflammatory factors such as VCAM-1, inhibition of activation of platelets, and inhibition of the migration of vascular smooth muscle cells [21].

The progression of arterial stiffness decreases vascular compliance and leads to arteries stenosis, eventually resulting in lower limb ischemic lesion [22]. Being another important pathogenic mechanism of PAD, arterial stiffness is a key target of clinical intervention. MAC is a nonobstructive condition leading to decreased arterial compliance, which has a strong independent predictor of total and cardiovascular mortality [23]. Although the adverse effects of MAC in the aorta on stiffening, systolic blood pressure, and coronary perfusion are well studied, the pathological effect of MAC in other vascular beds is poorly understood [24]. Rather than just simple precipitation of calcium and phosphate, the development of MAC is an active, cell-regulated process driven primarily by vascular smooth muscle cells (VSMCs) [25]. In the progression of MAC, the VSMCs were damaged and overburdened by hostile conditions in the microenvironment. As a result, those cells subsequently lost their essential defensive mechanisms and underwent transdifferentiation to promote an osteo/chondrogenic phenotype, which eventually drove the mineralization [24, 26]. To the best of our knowledge, the influence of BPS on MAC prevention has not yet been investigated. In the present study, patients in combined therapy group experienced markedly improvement of MAC in dorsal pedal artery at the end of the follow-up, when compared to the control group. We propose that BPS combined with aspirin might have an effect on inhibiting MAC progression, but further study is needed for the exploration of the underlying mechanism.

PWV is another important index used to evaluate the stiffness of arteries. It was reported that BPS has a beneficial effect on reducing the PWV in patients with T2DM [27]. In the present study, both groups did not differ significantly in the changes of the PWV at the end of the follow-up when compared to the baseline. As PWV was largely influenced by age, gender, blood glucose, serum lipid, blood pressure, and ABI, we suggested that the variation of the demographic data in different studies might be a reasonable explanation of the different results.

Aspirin is an inhibitor of COX-1, which has the effect of antiplatelet by blocking the cascade reaction of arachidonic acid and by preventing the production of TXA2 [28]. As we mentioned before, BPS binds with PGI2 receptor and has a potent cAMP-mediated inhibitory effect on platelet aggregation. The mechanisms of aspirin and beraprost sodium are different, and a previous study has proven that combined therapy of these two drugs would not increase the risk of bleeding [29]. In the present study, the combined therapy of BPS and aspirin did not increase the risk of hemorrhage and other adverse events, such as cardiovascular events and gastrointestinal adverse, when compared to the aspirin group. Thus, we suggested that the combined therapy of BPS and aspirin is safe.

This study was subjected to some limitations. Firstly, it was a single-center study with a small scale. Nonetheless, the findings are compelling and consistent with previous reports and the demographic data of the baseline and the last visit were comparable between the two groups. Correspondently, we believed that the conclusion of the present study is rather reliable. Secondly, patients were randomly assigned to treatment groups, but they were not treated in a blinded manner. Furthermore, we didn’t provide the specific PAD stage of each patient as well as the data of Ca and P. Lastly, we could not create a blank control group, as it was rejected by the institutional review board of our hospital. The exact effect of BPS on the prevention of arteries occlusion and stiffness in T2DM patients with PAD might be confounded by aspirin.

## Conclusion

Combined therapy of BPS and aspirin showed a protective effect on arteries occlusion and stiffness in patients with T2DM, with significant improvement of inner artery diameter and MAC in lower limbs. The combined therapy was also safe in those patients with favorable tolerance. Future study is needed for further exploration of the exact mechanisms.

## Data Availability

The datasets used and/or analyzed during the current study are available from the corresponding author on reasonable request.

## Abbreviations

PAD: Peripheral arterial disease
T2DM: Type 2 diabetes mellitus
BPS: Beraprost sodium
PGI2: Prostacyclin
MAC: Medial arterial calcification
CIMT: Carotid intima-media thickness
PWV: Pulse wave velocity
VSMCs: vascular smooth muscle cells
